# Supplementary material for: The Knockdown of TREK-1 in Hippocampal Neurons Attenuate Lipopolysaccharide-Induced Depressive-Like Behavior in Mice
Source: Int J Mol Sci. 2019 Nov 24;20(23):5902. doi: 10.3390/ijms20235902 (PMC6929152; doi:10.3390/ijms20235902)
Supplement: Supplementary file 1 [file ijms-20-05902-s001.pdf]

Supplementary Materials

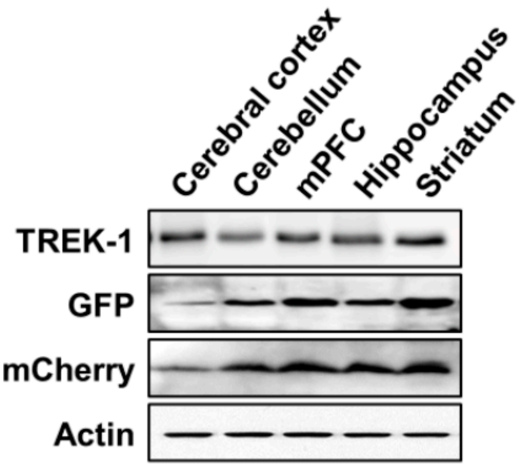

Figure S1. Regional expression of fluorescent protein in pSico-Red-shTREK-1 mice. (A) Western blot analysis from several brain tissues. Regional expression of TWIK-related potassium channel-1 (TREK-1), green fluorescent protein, and mCherry in pSico-Red-shTREK-1 mice.

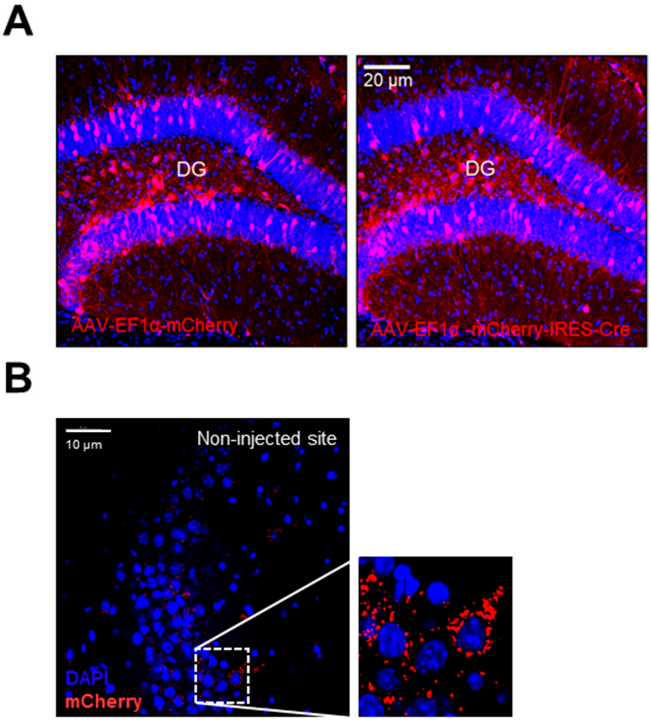

Figure S2. Confirmation of virus injection site by mCherry expression in pSico-Red-shTREK-1 mice. (A) Representative images showing the injection of adeno-associated virus (AAV)-elongation factor 1 alpha (EF1α)-mCherry or AAV-EF1α-mCherry-IRES-Cre in the dentate gyrus. (B) Non-injected site showed a low-intensity mCherry signal.

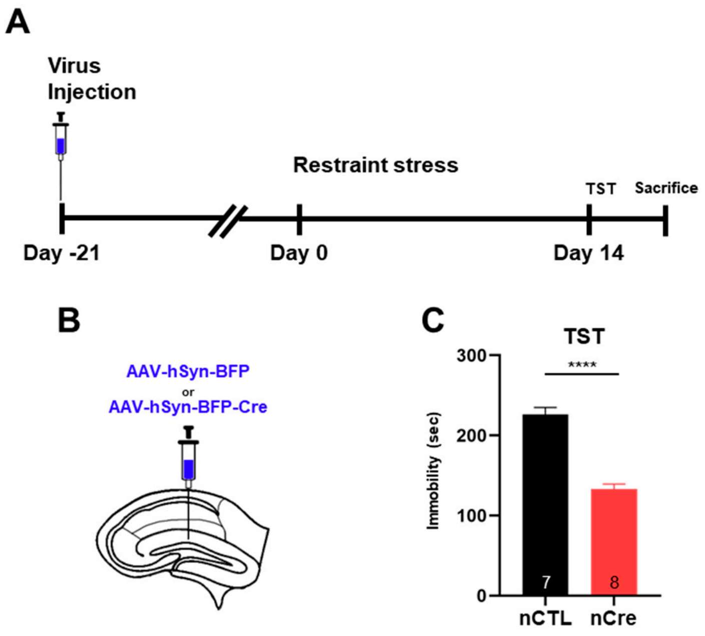

Figure S3. Tail suspension test (TST) in TWIK-related potassium channel-1 (TREK-1) conditional knockdown (cKD) mice after restraint stress. Experimental procedure for the restraint stress test schedule. Viruses were injected into the bilateral dentate gyrus, followed by a 21-day recovery (Day -21). Restraint stress was administered for 14 days. Fourteen days later, TST was conducted. (B) An illustration of a hippocampal slice of TREK-1 cKD mice to show the site of AAV-hSyn-BFP or AAV-hSyn-BFP-Cre injection. (C) Immobility time from the TST on mice after restraint stress. The numbers inside each bar indicate the number of samples. Data are presented as means ± standard error of the mean (*****p* < 0.0001).

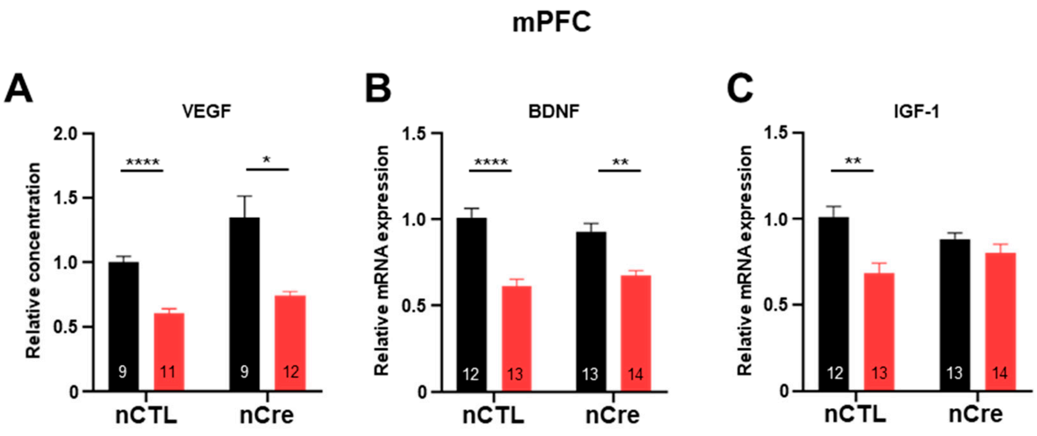

Figure S4. Quantitative real-time polymerase chain reaction analysis of neurotrophic factors in non-injected brain region (medial prefrontal cortex, mPFC). (A) Enzyme-linked immunosorbent assay for the vascular endothelial growth factor in mice (mPFC slice). Messenger ribonucleic acid levels of (B) brain-derived neurotrophic factor and (C) insulin growth factor-1. The numbers inside each bar indicate the number of samples. Data are presented as means ± standard error of the mean (**p* < 0.05, ***p* < 0.01, *****p* < 0.0001).
